# Supplementary material for: Beyond nothingness in the formation and functional relevance of voids in polymer films
Source: Nat Commun. 2024 Apr 11;15:2852. doi: 10.1038/s41467-024-46584-2 (PMC11009415; doi:10.1038/s41467-024-46584-2)
Supplement: Supplementary file 1 — Supplementary Information [file 41467_2024_46584_MOESM1_ESM.pdf]

Supplementary Information for

Beyond Nothingness in the Formation and Functional Relevance of Voids in Polymer Films

Falon C. Kalutantirige *et al.*

*Corresponding authors Email: yli2562@wisc.edu and qchen20@illinois.edu

This file includes:

Supplementary Notes 1 to 4
Supplementary Figs. 1 to 18
Supplementary Tables 1 to 7
Legends for Supplementary Movies 1 to 4
Supplementary References

Other Supplementary materials for this manuscript include the following:

Supplementary Movies 1 to 4

Supplementary Note 1: Synthesis and nanomorphology characterisation of control PA membranes

Control PA membranes were synthesised without the supporting PS substrate layer, with similar starting monomer concentrations to the PA membranes under study. Thus, control PA1, control PA2 and control PA3 were prepared with 0.05, 0.1 and 0.1 w/v% c_{TMC} and a constant c_{MPD} of 5 w/v%. As evident from the two-dimensional (2D) TEM and three-dimensional (3D) electron tomographic images (Supplementary Fig. 3), the control PA membranes show an interconnected crumpled morphology, with distinct crumple densities and crumple sizes for each starting monomer condition. Similar to their counterparts synthesised in the presence of PS substrate (i.e. PA1, PA2 and PA3), the crumple density of the control membranes increases as c_{TMC} increases. The crumple walls of the control membranes are interconnected and the membranes have nanovoids spanning the 3D space (Supplementary Fig. 3). The degree of crosslinking (DOC) and elemental composition of the control PA membranes are comparable to those of the PA membranes under study, with DOC following the same trend (control PA1 > control PA2 > control PA3) (Supplementary Fig. 3, Supplementary Table 3).

Supplementary Note 2: Previous work on atomistic and coarse-grained molecular dynamics simulations (CGMD) of polyamide membranes

Previous simulation efforts on PA membrane formation are mostly based on atomistic models. In a study conducted by Kolev *et al.*[1], atomistic PA membranes were generated to provide insights into the mechanisms involved in the formation, hydration, and functioning of membranes during the interfacial polymerization (IP) processes. To simulate this, they initially set up a simulation box containing MPD and TMC monomers, along with a few TMC/MPD dimers acting as initial clusters. Following the initial setup, the monomers in the simulation were allowed to react exclusively with the growing clusters, while preventing reactions between monomers. With the formation of each new bond, a monomer of the same type as the one that reacted was introduced into the simulation box. This introduction occurred randomly within the vacant space not occupied by the van der Waals volume of the polymeric clusters. This simulation procedure closely resembles the stoichiometrically balanced diffusion of monomers from adjacent solutions into the reaction zone, as observed in IP processes, although the size of the simulation box is too small to capture nanomorphology.

Li *et al.*[2] performed equilibrium molecular dynamics (EMD) simulations to simulate the construction process of PA membranes at an atomic level. They investigated the morphological transformation and mass transport through the polymer network by varying the stoichiometry of the monomers. In their study, MPD and TMC molecules were initially placed in a cubic cell using a heuristic algorithm to ensure randomization. To achieve crosslinking between MPD and TMC, they utilised an update to the reaction radius. Specifically, if the nitrogen atom in a free amine group came within 3.25 Å of a carbonyl carbon atom in a free acyl chloride group, a molecular topology transformation was triggered. This transformation involved the formation of an amide bond while simultaneously removing any excess hydrogen and chlorine atoms present. The crosslinking process involved continuous updates to the atomic radii of the reaction until reaching either the maximum C-N cutoff distance or the desired conversion target. Different MPD:TMC

ratios were considered, corresponding to different stoichiometric ratios based on the amine:acyl chloride functional group molar ratio.

He *et al.*[3] performed non-equilibrium molecular dynamics (NEMD) simulations to investigate the effect of different manufacturing methods on the performance of PA membranes. For IP in particular, a fixed 3:2 MPD:TMC ratio was used for the simulations. The reacted atoms were identified and arranged into TMC and MPD monomers. Each monomer type provided specific potential reaction sites. These monomers were then filled into separate 3D-periodic cells of equal cross-section size. The two cells were assembled along the direction normal to the cross-section, creating a composite with an interfacial layer between the MPD and TMC layers. The cross-linking reaction occurred randomly and was restrained to the MPD/TMC interface, simulating the IP process.

In a study by Shen et al.[4], NEMD simulations were used to study the atomic-scale transport of water, ions, and small organic solutes in a commonly used membrane. In this process, the TMC and MPD monomers were randomly moved in a computational box. When the functional groups of the monomers responsible for cross-linking were within a specified distance from each other, an amide bond was formed, subsequently building the polymeric structure. The aim was to mimic the variability observed in actual polymerization processes, considering the inherent randomness involved.

Supplementary Note 3: CGMD simulations of varying monomer diffusion rates

We incorporated three additional simulations to investigate the impact of MPD diffusion rates on stoichiometry within the reaction zone and the final PA membrane density. To optimise computational efficiency, we utilised the model and simulation setup previously established by Muscatello *et al*.[5]. The simulation box dimensions are (10 × 10 × 200) nm^3, with a TMC:MPD concentration ratio of 1:1. Subsequently, to characterise three distinct monomer diffusion rates, we arbitrarily varied the mass input for the MPD monomer as 0.3, 1.0, and 3.0 times the original molecular mass[6]. Note that the diffusion rate of MPD monomers in hexane[7] is 10^{-6} cm^2 s^{-1}, while in our CGMD simulations, the diffusion rate of CG-MPD monomers is 1.265×10^{-6} cm^2 s^{-1}, a result of using implicit solvent conditions. We observed that diverse diffusion rates affect the local membrane configuration during the reaction process, resulting in denser membrane structures when the rate of diffusion of MPD monomer is low and less dense membrane structures with a higher rate of diffusion compared to the original monomer diffusion rate. In other words, the different diffusion rates of the amine monomers affectthe monomer stoichiometry in the reaction zone as observed with the distinct local membrane configuration (Supplementary Figs. 11,12).

Supplementary Note 4: Derivation of solvent permeance fittings

We used the Spiegler-Kedem model as the transport equation for the permeance fitting as shown in equation (1) below, where J_v is the volumetric flux, L_p is the hydraulic permeance, ΔP is the transmembrane pressure, σ is the reflection coefficient and Δπ is the osmotic pressure.

$$J_v = L_p(\Delta P - \sigma\Delta\pi) \quad (1)$$

In the Spiegler-Kedem model, L_p is given by the solvent-membrane permeability (P_m) and the thickness of the membrane (δ), which is used as the basis to derive our fitting (equation (2)).

$$L_p = \frac{P_m}{\delta} \quad (2)$$

To determine the nominal membrane thickness, the membrane was categorized into three distinct regions, which are depicted in Supplementary Fig. 13. Region 1 is the thin open void, region 2 is the featureless base layer, and region 3 is the thick closed void section.

Tomographic reconstructions were used to calculate the top (A_T) and bottom (A_B) surface areas of the PA membrane, the surface area of open voids (A_{OV}) and the surface area of closed voids (A_{CV}). The geometric area (A_G) is the projection area of the tomographic reconstruction (Supplementary Table 5).

Two approaches are used to fit the experimental methanol permeance. The first fitting (Approach 1) used open and closed void areas, and the local membrane thicknesses measured using tomographic reconstruction.

For Approach 1, the surface areas of region 1 and region 3 are given by A_{OV} and A_{CV} respectively, and the surface area of the featureless region 2 (A_F) is given by equation (3).

$$A_F = A_T - (A_{OV} + A_{CV}) \quad (3)$$

Using the surface area data from tomography, the area percentage for each region as shown in equations (4), (5) and (6) where region 1, region 2 and region 3 are represented by $\%A_{open}$, $\%A_{flat}$ and $\%A_{closed}$, respectively.

$$\%A_{open} = \frac{A_{OV}}{A_{OV} + A_{CV} + A_F} \quad (4)$$

$$\%A_{flat} = \frac{A_F}{A_{OV} + A_{CV} + A_F} \quad (5)$$

$$\%A_{closed} = \frac{A_{CV}}{A_{OV} + A_{CV} + A_F} \quad (6)$$

Three assumptions are driven by observations from the 3D tomographic reconstructions of PA membranes, thickness mapping and void reconstructions; (i) open void regions are surrounded by t_1 and t_2 thickness, (ii) closed void regions are enclosed by one layer of t_2 thickness and another layer of t_3 thickness at the base, and (iii) the flat membrane region is of t_2 thicknesses.

Using the above assumptions, the nominal open void thickness (region 1), δ_{open}, was estimated using the multimodal thickness maxima from tomography (t_1, t_2, t_3) and their weighted percentages (x_1, x_2, x_3) as shown in equation (7).

$$\delta_{\text{open}} = \frac{x_1}{x_1 + x_2} * t_1 + \frac{x_2}{x_1 + x_2} * t_2 \quad (7)$$

To estimate the nominal closed void thickness (region 3), δ_{closed}, the thickness maxima enclosing the top void region, t_3, was added to the thickness maxima representing the bottom surface thickness t_2 (equation (8)).

$$\delta_{\mathrm{closed}} = t_2 + t_3 \tag{8}$$

The flat featureless layer (region 2) was estimated with the thickness maxima t_2 (equation (9)).

$$\delta_{\mathrm{bottom}} = t_2 \tag{9}$$

To calculate the nominal thickness ($\delta_{\mathrm{nom,1}}$) from these three distinct regions, a harmonic mean of each region's thickness weighted by the percentage of area of that region was used as shown in equation (10).

$$\delta_{\mathrm{nom,1}} = \left(\frac{\%A_{open}}{\delta_{open}} + \frac{\%A_{closed}}{\delta_{closed}} + \frac{\%A_{bottom}}{\delta_{bottom}}\right)^{-1} \tag{10}$$

The second fitting (Approach 2) used membrane thicknesses derived from AFM measurements ($\delta_{\mathrm{nom,2}}$) as shown in Supplementary Fig. 14.

For both approaches, the permeability (P_{m}) was then calculated given the nominal membrane thickness and experimentally determined solvent permeance ($L_{\mathrm{exp,k}}$) with the following equation:

$$P_{\mathrm{m,k}} = L_{\mathrm{exp,k}} \times \delta_{\mathrm{nom,k}} \tag{11}$$

The material properties of the membranes were assumed to be dependent on the mass. A permeability constant (μ) was defined to isolate the effect of polymer density (equation (12)), as the solvent-membrane permeability is inversely proportional to membrane density. Note that the density is a linear function of the degree of crosslinking within our range of interest (41% < DOC < 98%) with an R^2 of 0.98, thus the density was calculated using DOC based on previous work[8,9].

$$\mu_{\mathrm{k}} = P_{\mathrm{m,k}} \times \rho_{\mathrm{k}} \tag{12}$$

The permeability constant for each PA membrane was averaged to form an average permeability constant (μ_{avg}), and used to calculate the solvent permeance ($L_{\mathrm{fit,k}}$) as follows (equation (13)).

$$L_{\mathrm{fit,k}} = \frac{\mu_{\mathrm{avg}}}{\rho_{\mathrm{k}} \times \delta_{\mathrm{nom,k}}} \tag{13}$$

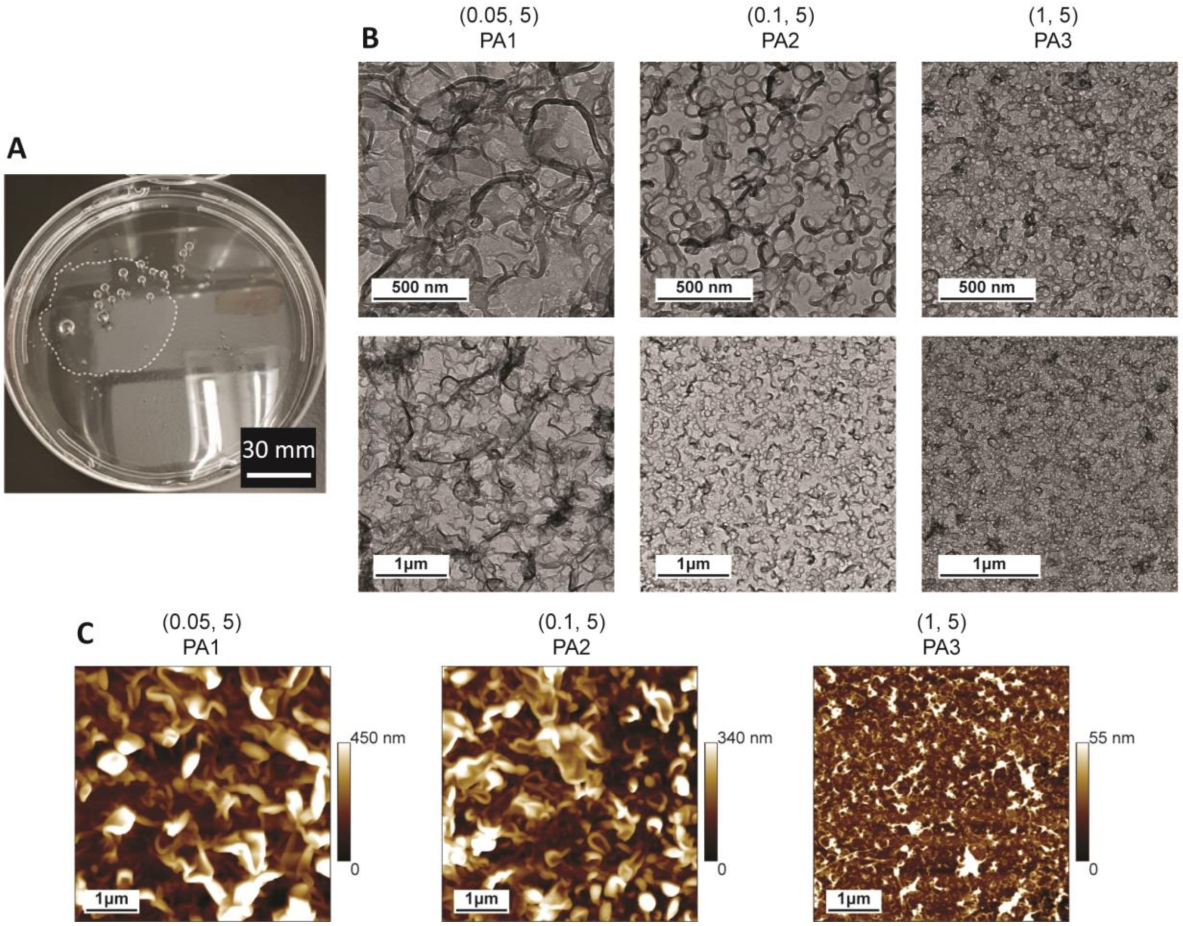

Supplementary Fig. 1. TEM images and AFM height maps of PA membranes with interconnected voids and crumples synthesised at three different starting monomer concentrations (c_{TMC}, c_{MPD}). (**A**) Photograph of a freestanding PA membrane floating on the air–water interface. The membrane is outlined using white dashed lines. (**B**) TEM micrographs of PA1, PA2 and PA3 at two different magnifications show the morphology differences between the three systems. (**C**) AFM height maps of PA1, PA2 and PA3 show the membrane height variations.

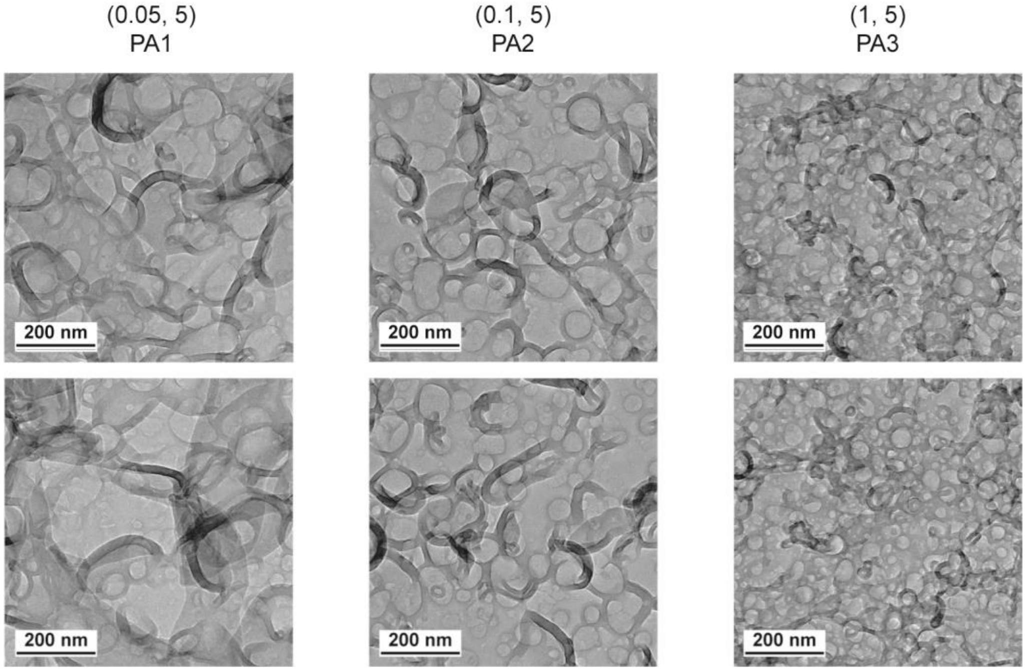

Supplementary Fig. 2. TEM projections at 0° tilt of the three PA membrane synthesis conditions. These projection regions were used for tomographic reconstruction. Two samples per system were used for 3D morphology analysis to show morphological similarities.

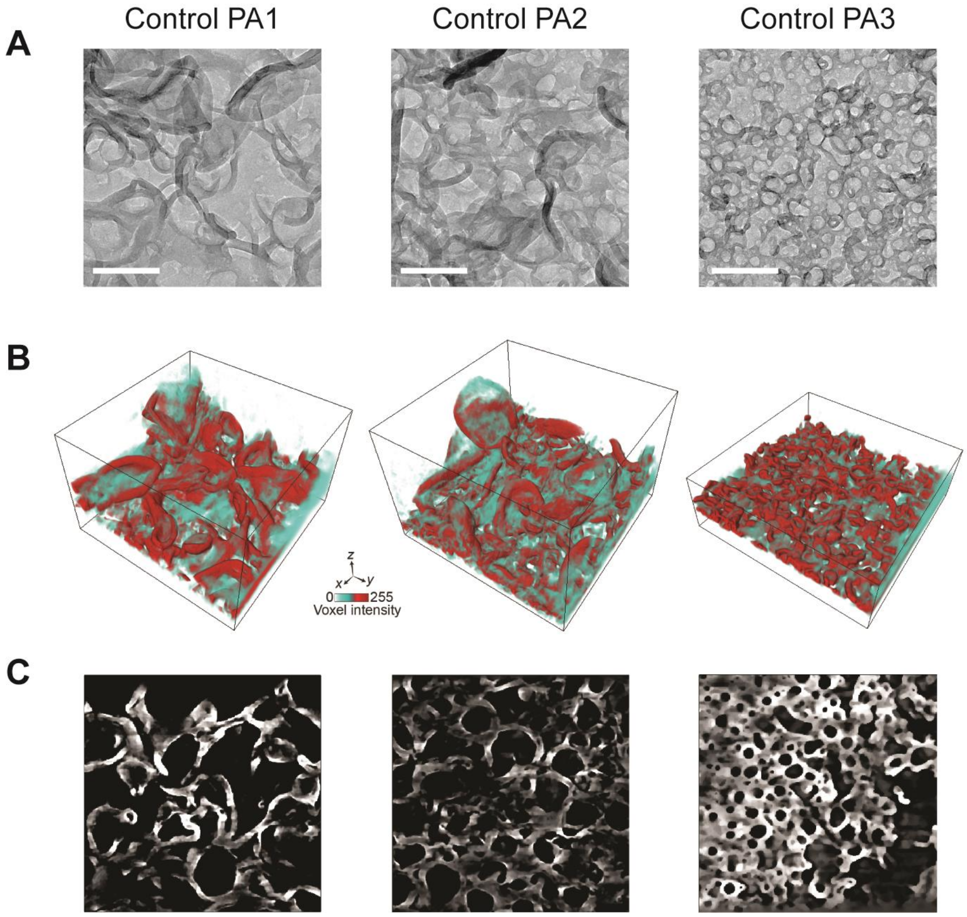

Supplementary Fig. 3. TEM imaging and 3D tomographic reconstructions of control PA membranes synthesised without supporting substrates. (**A**) TEM images of control PA1, control PA2 and control PA3 membranes synthesised without the PS support layer. Note that the starting monomer concentrations for control PA1, control PA2 and control PA3 are c_{TMC}= 0.05, 0.1 and 1 w/v%, respectively, and c_{MPD}= 5 w/v% for all. (**B**) Grayscale intensity-based electron tomographic reconstructions of control PA membranes showing an interconnected crumpled morphology similar to their counterparts synthesised in the presence of PS support layer. The projection area of reconstruction is 727 nm × 727 nm, and the reconstruction height varies with the crumple heights. (**C**) The bottom *xy*-slices of 727 nm × 727 nm for the three membranes showing inner void regions and interconnected crumple walls. Scale bars: 200 nm.

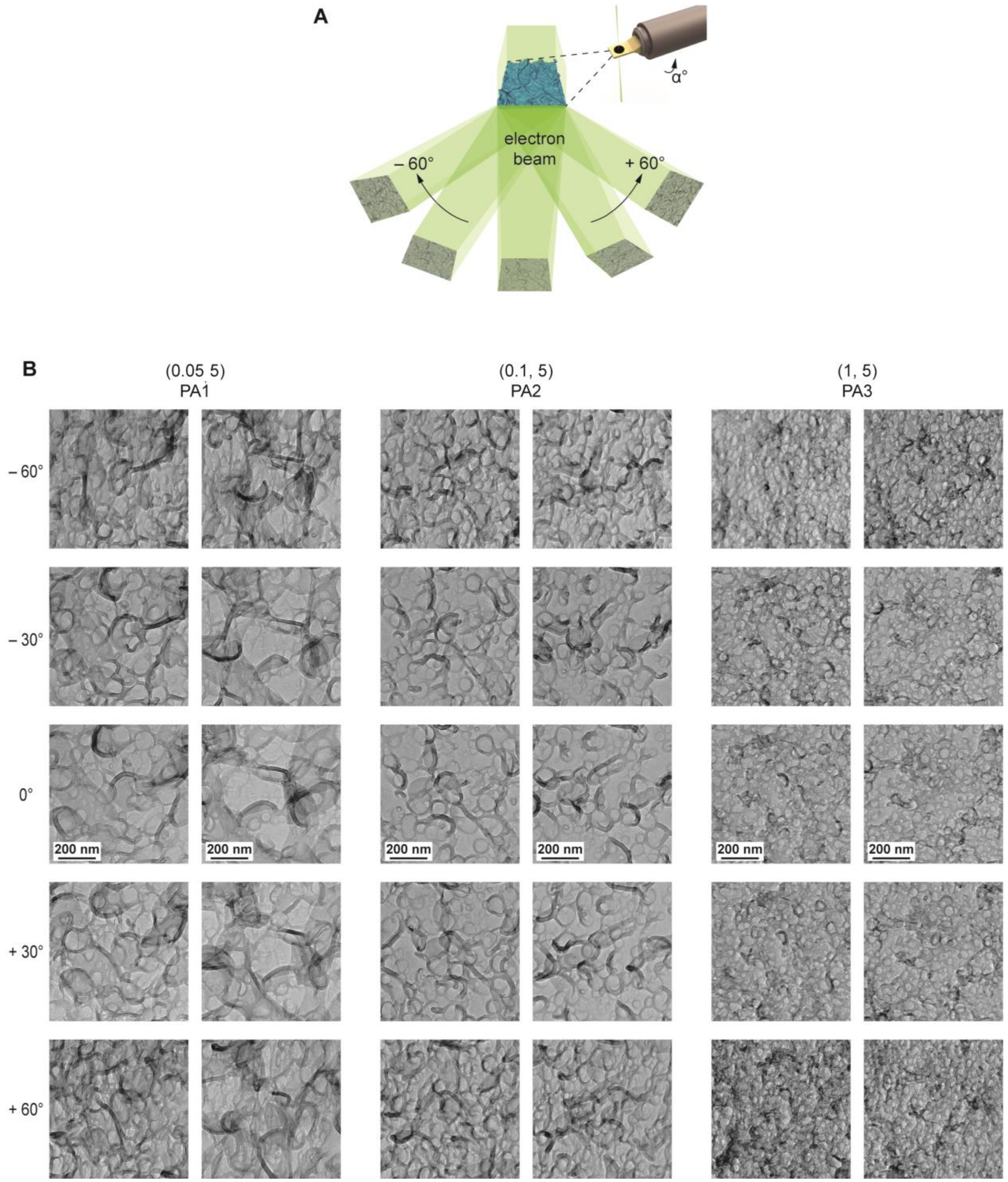

Supplementary Fig. 4. Electron tomography set-up and selected TEM images from tilt series. (**A**) Schematic showing electron tomography set-up with tilt angle α and tilt range. (**B**) Selected tilt TEM micrographs of networked PA membranes.

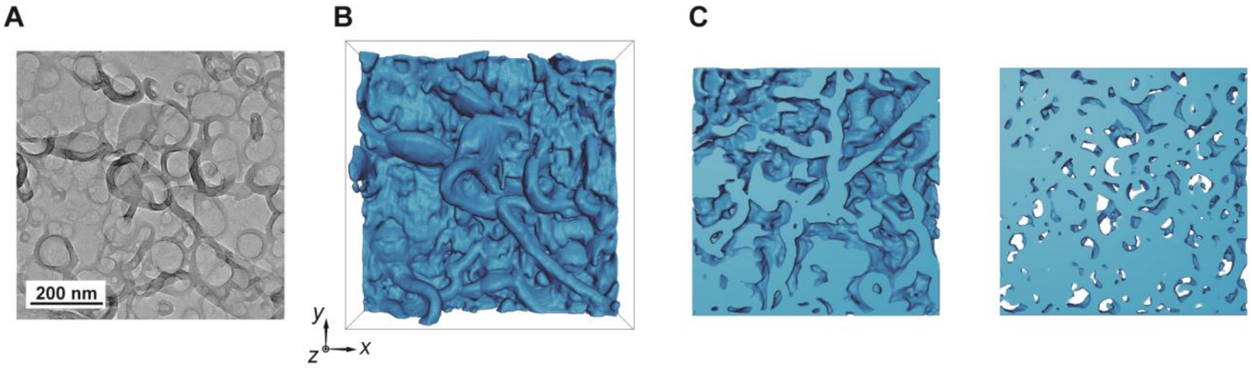

Supplementary Fig. 5. Tomographic reconstruction shows complex networked crumple morphology of PA membranes. TEM micrograph (**A**) and 3D tomographic reconstruction (**B**) of PA membrane. (**C**) Two volume slices at different *z*-heights of the 3D reconstruction in (**B**) showing the internal nanomorphology with voids and varying local thickness, which is not captured in (**A**).

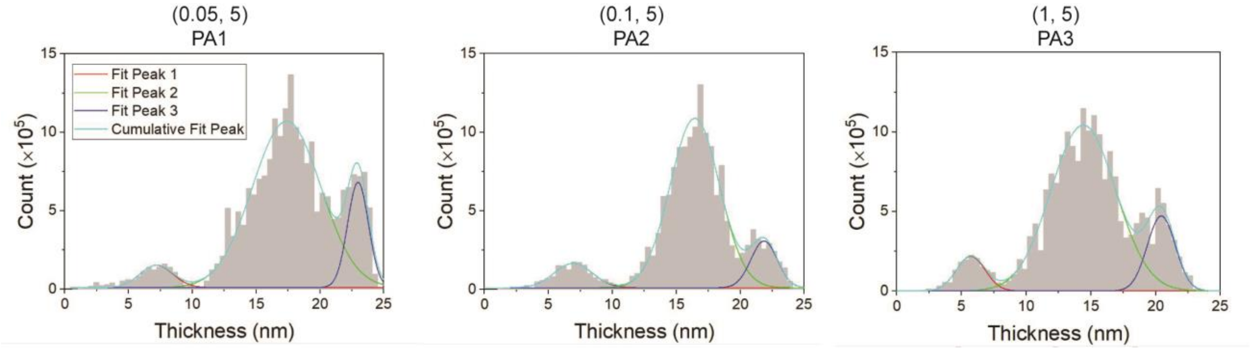

Supplementary Fig. 6. Gaussian fitting of multimodal peaks to calculate local thickness maxima. Peak maxima and fitting parameters are given in Supplementary Tables 1 and 2.

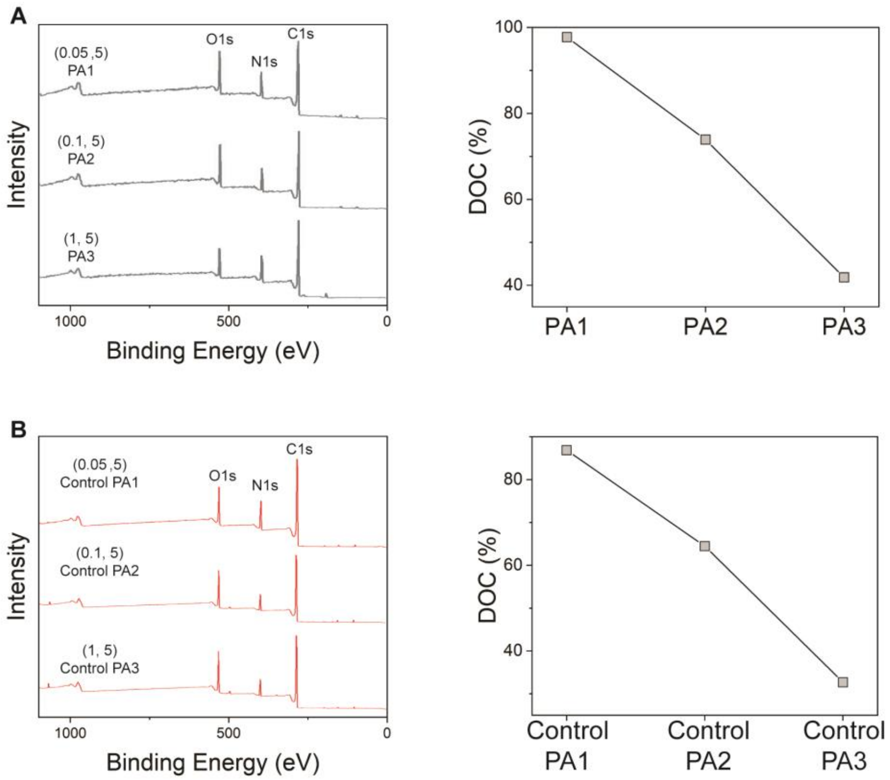

Supplementary Fig. 7. Characterisation of crosslinking density of PA membranes. XPS spectra of PA and DOC presented as a function of monomer concentration ratios for (**A**) PA membranes under study and (**B**) control PA membranes as a comparison.

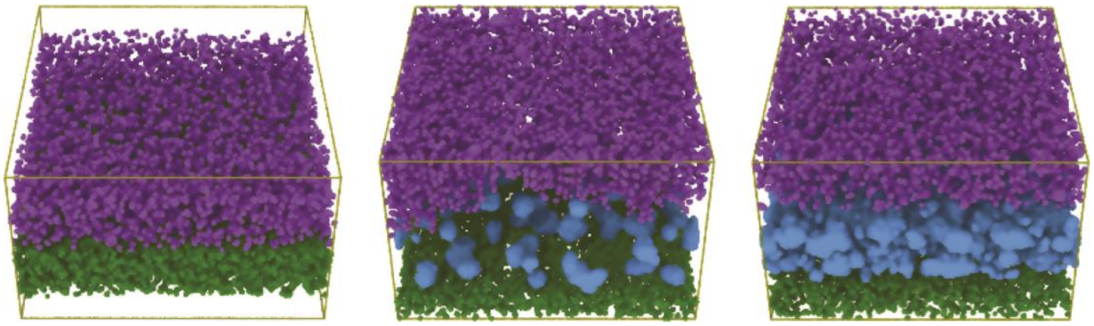

Supplementary Fig. 8. MD simulation for PA membrane with 1:4 TMC:MPD ratio (PA4). The snapshots provided illustrate the formation of a spanning membrane through a CGMD simulation at three successive times: 0 ns, 90 ns, and 600 ns, displayed from left to right.

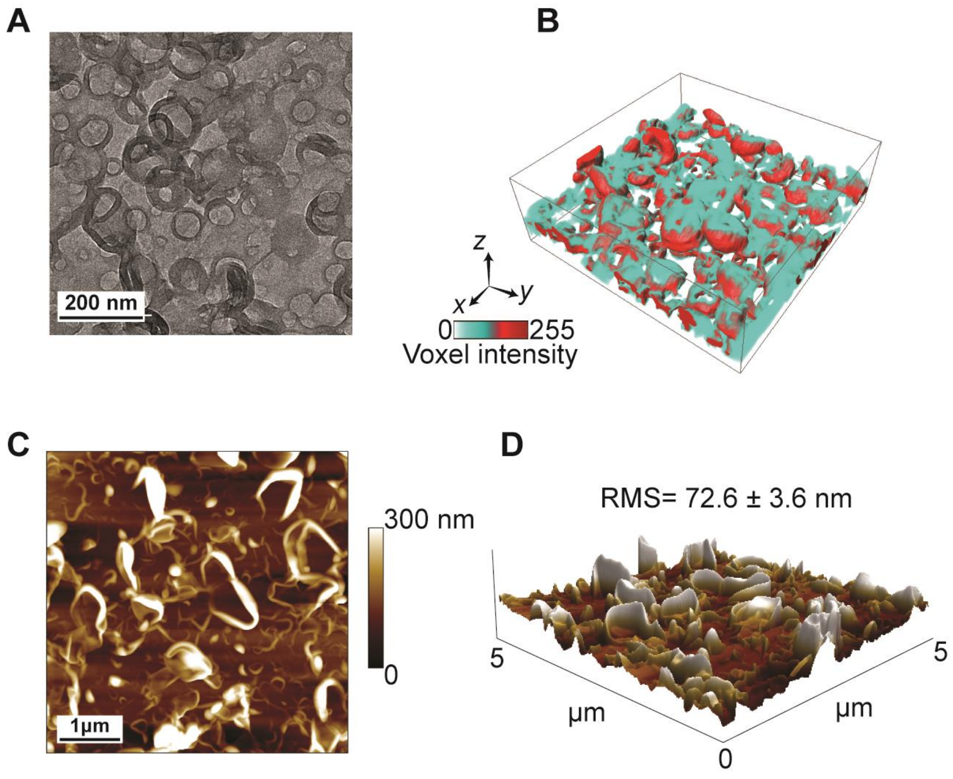

Supplementary Fig. 9. Tomographic reconstruction and AFM maps of PA4 (2 w/v% c_{TMC} and 3.26 w/v% c_{MPD}). (**A**) TEM image of PA4 showing networked crumple morphology. (**B**) Tomographically reconstructed PA4 membrane region. AFM height map (**C**) and image showing RMS roughness (**D**).

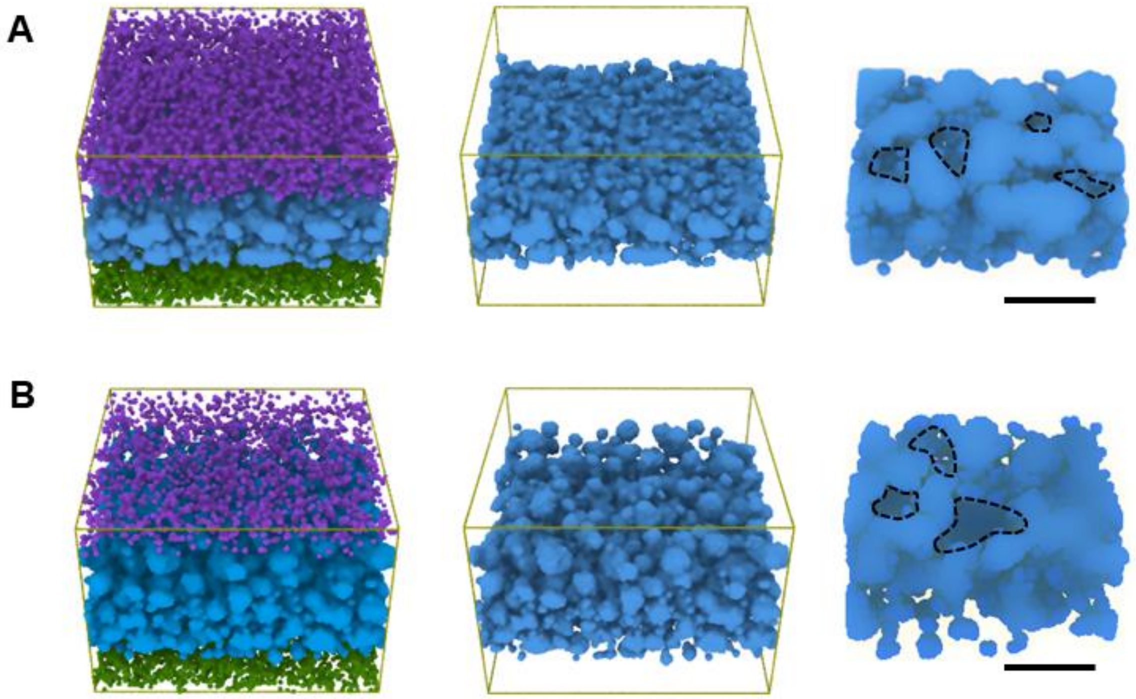

Supplementary Fig. 10. Nanovoids visualized in CGMD simulations. Snapshots of CGMD simulations of PA membranes with TMC:MPD monomer ratios of (**A**) 1:4 (PA4) and (**B**) 1:12.3 (PA3) after 600 ns. Snapshots of PA membrane with TMC (purple) and MPD (green) monomers (left), PA membrane without monomers to show the PA oligomer clusters (middle), and a zoomed-in view of the nanovoids (right). Note that the nanovoids are outlined using dashed lines. The box dimensions are (100 × 130 × 100) nm3. Scale bars: 4 nm.

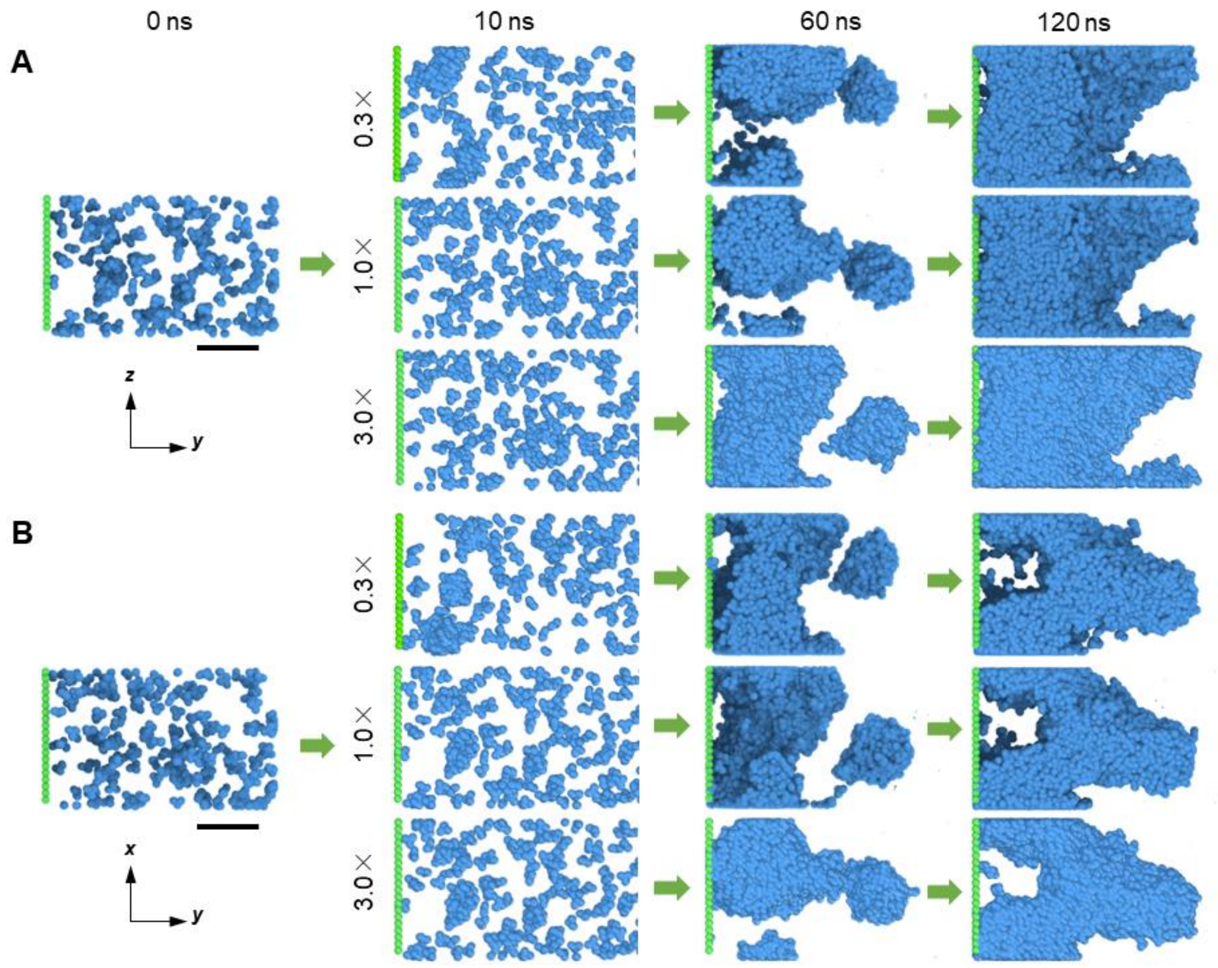

Supplementary Fig. 11. Effect of monomer diffusion rate on PA morphology characterised by CGMD. For a starting monomer ratio of 1:1 TMC:MPD, snapshots illustrate the evolution of a spanning membrane in a CGMD simulation in both (**A**) yz plane and (**B**) xy planes at four consecutive time points: 0 ns, 10 ns, 60 ns, and 120 ns. The molecular mass of MPD was varied to simulate three distinct rates of diffusion, as 0.3 times the original monomer mass (top), the original monomer mass (middle), and 3 times the original monomer mass (bottom). The snapshots are sequentially arranged from left to right, providing a visual representation of the successive stages of membrane formation over time. Scale bars: 5 nm.

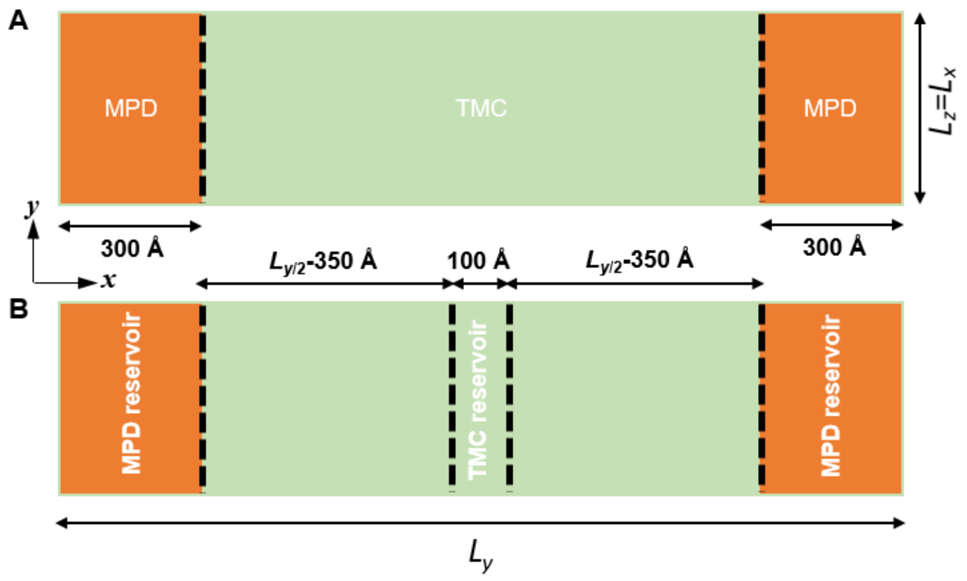

Supplementary Fig. 12. CGMD setup for diffusion rate simulations. (**A**) The initial distribution depicts TMC (light green) and MPD (orange) monomers within the simulation cell before initiating IP reactions. Grids of repulsive potentials, denoted by black dashed lines, were employed to maintain separation between TMC and MPD monomers. (**B**) After the initial equilibration, the repulsive interactions between the black dashed lines and MPD monomers were disabled. This allows MPD monomers to diffuse from the orange region, where they were initially confined, into the light green region. TMC monomers remain confined to their original region, as shown in (A). The locations of reservoirs for MPD and TMC monomers are outlined by thin black dashed lines. Polymerisation reactions occur when MPD comes into contact with TMC. Periodic boundary conditions were applied to all faces of the simulation cell.

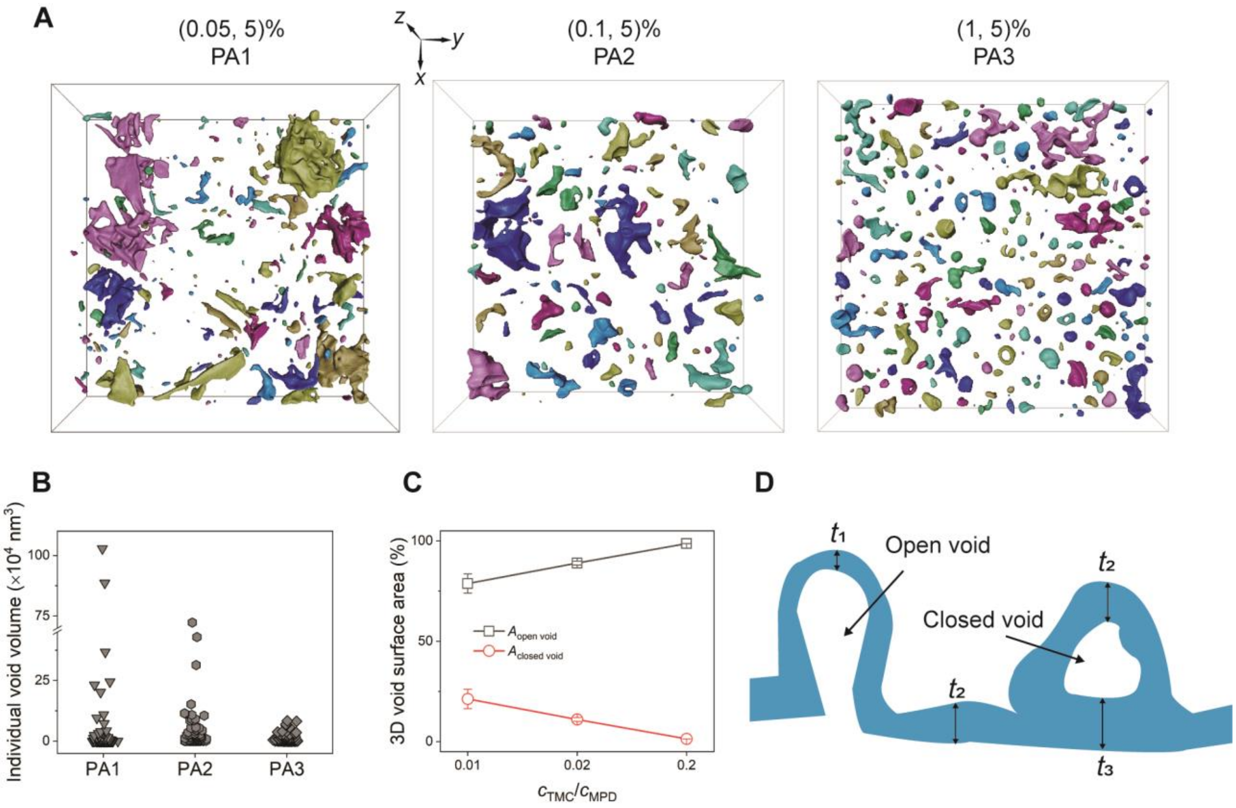

Supplementary Fig. 13. Void maps coloured to show separate individual void islands. (**A**) Void island regions for a second tomographic reconstruction of PA1, PA2 and PA3 in addition to those shown in Fig. 3d. (**B**) Individual void volume distributions for maps shown in (**A**). (**C**) Percentage surface areas of open and closed voids. The open and closed void surface area values are tabulated in Supplementary Table 5. (**D**) Schematic of thickness values observed from tomographic imaging used as assumptions in permeance modelling. It is assumed that open void surface areas are surrounded by t_1 thickness, and the closed void surface areas are enclosed by one layer of t_2 thickness and another layer of t_3 thickness at the base. The flat membrane region is of t_2 thicknesses. All assumptions were based on observations from 3D reconstructions.

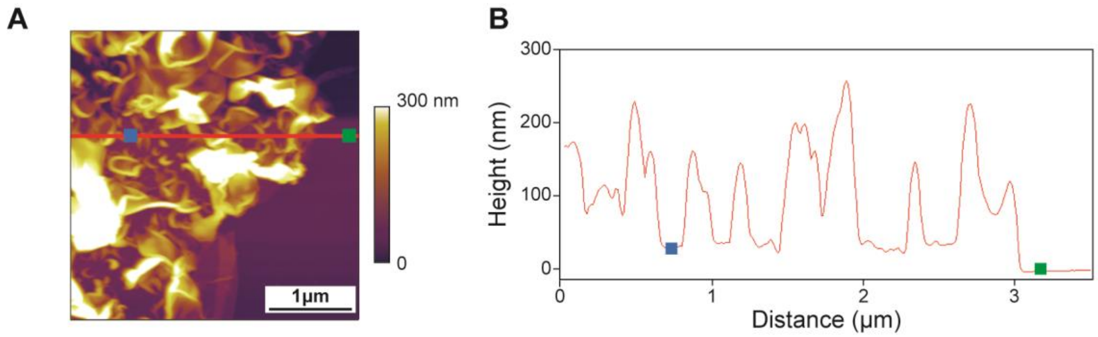

Supplementary Fig. 14. Membrane thickness measurement using AFM. AFM measures the membrane thickness from the height difference between the substrate (green square) and membrane (blue square).

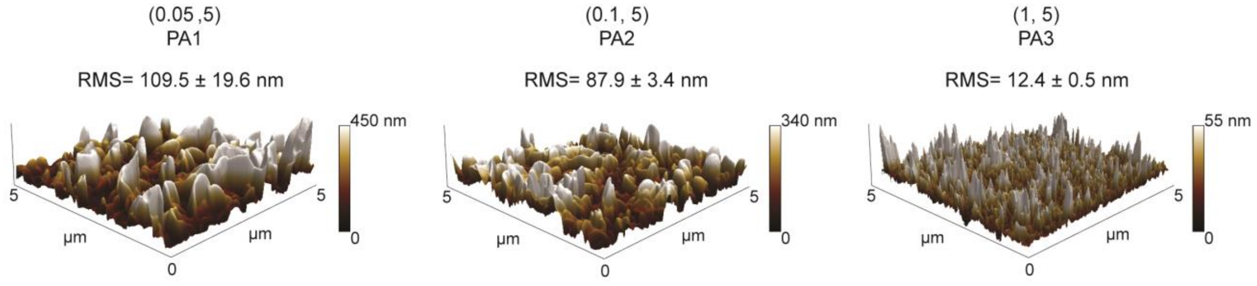

Supplementary Fig. 15. Root mean square (RMS) roughness of PA membranes. AFM measurements showing the RMS roughness of PA membranes from the three synthesis conditions.

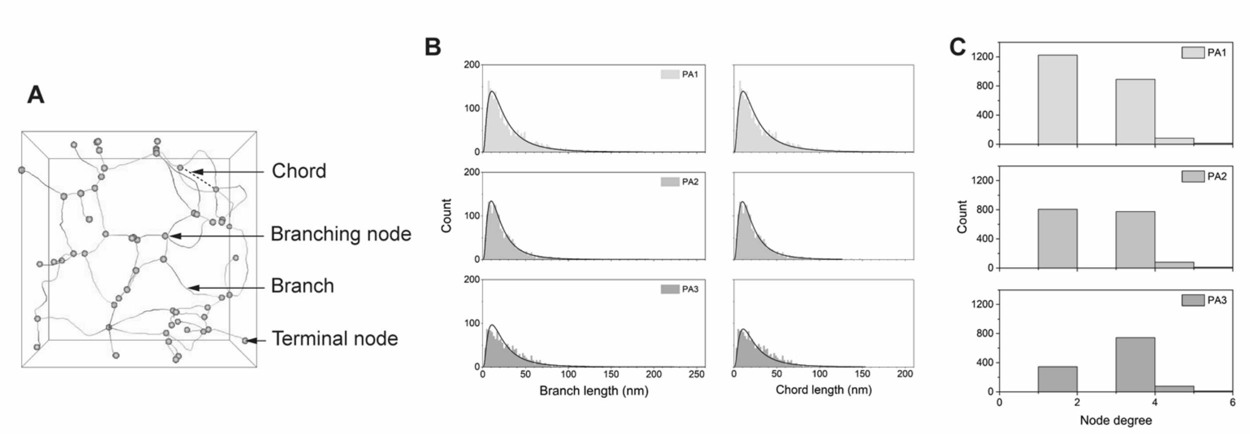

Supplementary Fig. 16. Components of a skeleton. (**A**) Schematic showing types of nodes, branch and chords (i.e., shortest distance between two nodes). (**B**) Branch length and chord length distributions for the three PA membrane skeletons. (**C**) The number of connected neighbours shown as the node degree. Note that there are no nodes with a degree of two (i.e. two connecting neighbours).

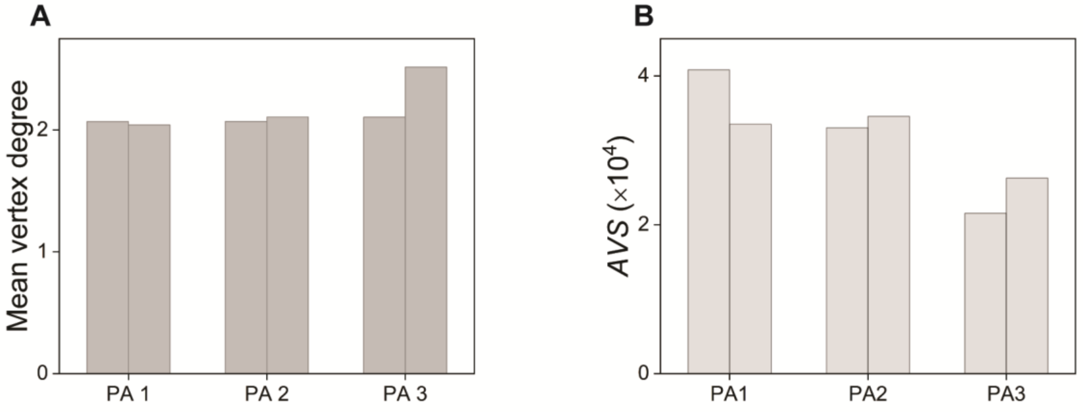

Supplementary Fig. 17. Additional graph theory (GT) parameters for PA membrane graph networks. (**A**) Mean vertex degree and (**B**) augmented vertex sum (AVS) parameters for the six PA membranes belonging to PA1, PA2 and PA3 synthesis conditions. Equations and calculations are given in Supplementary Table 7.

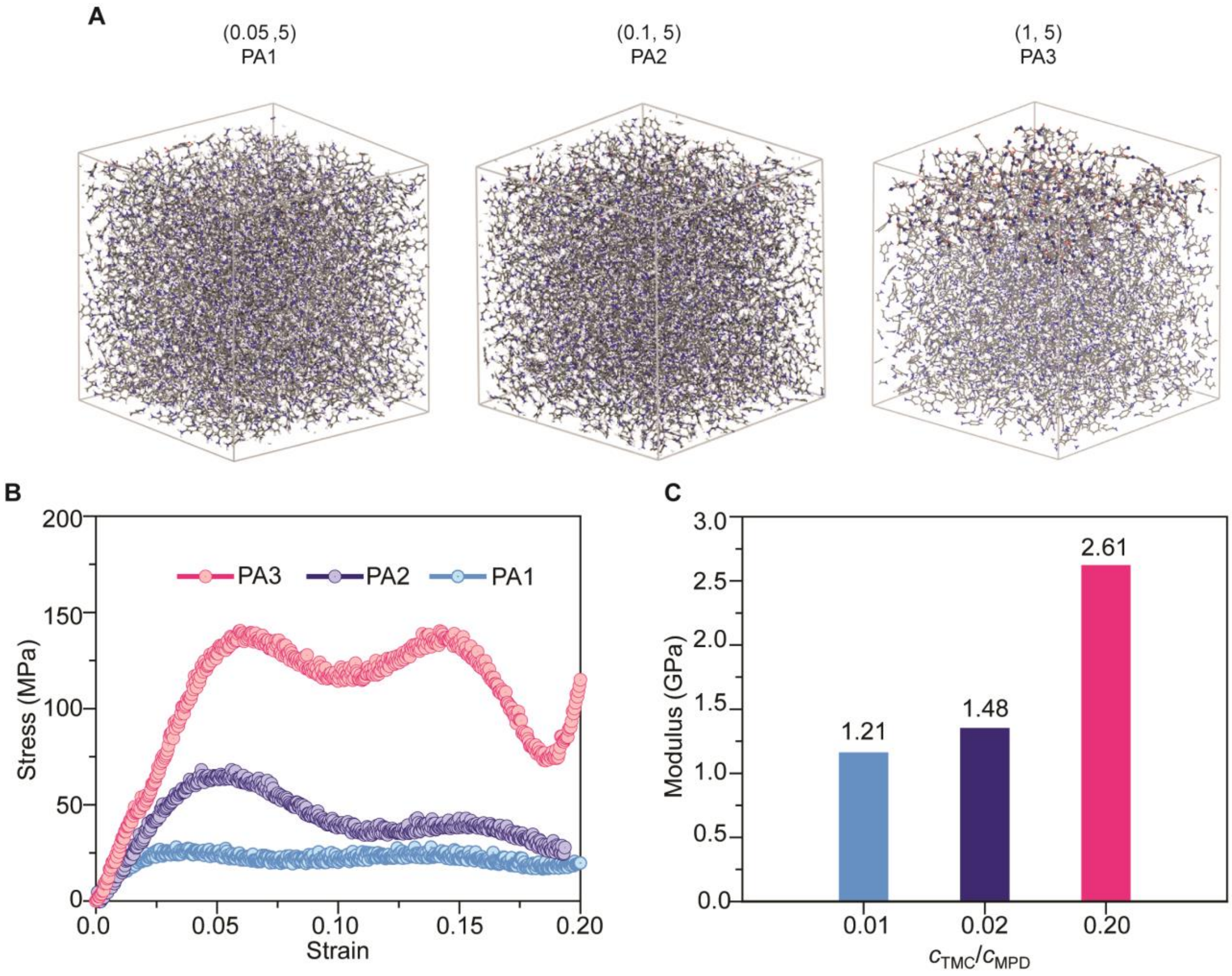

Supplementary Fig. 18. Models for stress-strain curves and modulus of PA membranes. (**A**) IP atomistic models for three different TMC/MPD ratios, PA1, PA2 and PA3, using experimental IP procedures. (**B**) Stress-strain curve of PA membrane with the three different TMC:MPD ratios under uniaxial compression loading, and (**C**) their corresponding modulus.

Supplementary Table 1. Gaussian model fitting equation and parameters for peaks in Supplementary Fig. 6.

$$\boldsymbol{y = y_0 + \frac{A}{w} \times \frac{\sqrt{\pi}}{4 \times \ln(2)} \times \exp\left(-4 \times \ln(2) \times \frac{(x - x_c)^2}{w^2}\right)}$$

	**PA1**		
	Peak 1	Peak 2	Peak 3
y_0	0.09953±0.277466	0.09953±0.277466	0.09953±0.277466
x_c	7.18275±0.48859	17.38199±0.10875	22.97328±0.08976
A	4.6192±2.33877	71.17988±4.33617	13.75912±1.72298
w	3.03473±1.36778	6.30982±0.33267	1.9279±0.22444
X^2	0.80888		
R^2	0.95007		

	**PA2**		
	Peak 1	Peak 2	Peak 3
y_0	0.10942±0.18021	0.10942±0.18021	0.10942±0.18021
x_c	6.93589±0.33484	16.43941±0.05941	21.85354±0.16253
A	5.77824±1.79791	52.11183±2.15443	7.67182±1.29284
w	3.4941±0.96207	4.54236±0.16991	2.42558±0.39827
X^2	0.39211		
R^2	0.96849		

	**PA3**		
	Peak 1	Peak 2	Peak 3
y_0	0.00534±0.2118	0.00534±0.2118	0.00534±0.2118
x_c	5.71359±0.2564	14.40761±0.0907	20.43102±1.7169
A	6.06374±1.6697	65.42302±3.1894	12.79783±1.7169
w	2.61355±0.6778	5.89517±0.26251	2.55168±0.32265
X^2	0.59481		
R^2	0.95622		

Supplementary Table 2. Thickness maxima values of the multimodal distribution of local thickness histograms for PA1, PA2 and PA3.

**Thickness maxima**	**PA1**	**PA2**	**PA3**
t_1 (nm)	7.2 ± 0.5	6.9 ± 0.3	5.7 ± 0.3
t_2 (nm)	17.4 ± 0.1	16.4 ± 0.1	14.4 ± 0.1
t_3 (nm)	23.0 ± 0.1	21.9 ± 0.2	20.4 ± 0.1

Supplementary Table 3. Atomic composition and DOC generated from XPS measurements for PA membranes synthesised with substrate and the control PA membranes synthesised without substrate (Supplementary Fig. 7).

	**Atomic composition from XPS (%)**			**DOC (%)**
	C	O	N	
PA1	74.80	12.70	12.50	97.7
PA2	75.51	13.31	11.18	73.9
PA3	76.58	13.98	9.44	41.8
Control PA1	76.91	12.05	11.04	86.88
Control PA2	77.54	12.56	9.90	64.47
Control PA3	78.83	12.96	8.21	32.69

Supplementary Table 4. Material and void volume parameters for PA1, PA2 and PA3.

**Volume parameter**	**PA1**	**PA2**	**PA3**
v_{tm} ($\times 10^{-3}$ μm^3)	49.2 ± 4.0	42.4 ± 5.7	32.4 ± 3.4
v_{tv} ($\times 10^{-3}$ μm^3)	12.7 ± 1.2	8.7 ± 0.9	6.1 ± 0.3
r (%)	25.7 ± 0.4	20.5 ± 0.6	19.0 ± 1.1
$f_{material}$ (%)	79.5 ± 0.2	83.0 ± 0.4	84.0 ± 0.8
f_{void} (%)	20.5 ± 0.2	17.0 ± 0.4	16.0 ± 0.8

Supplementary Table 5. The 3D surface areas of membrane top surface, bottom surface, geometric/projected area and open and closed voids extracted for permeance modelling.

	**Open void area** ($\times 10^{-2}$ μm^2)	**Closed void area** ($\times 10^{-2}$ μm^2)	**Top surface area** ($\times 10^{-2}$ μm^2)	**Bottom surface area** ($\times 10^{-2}$ μm^2)	**Geometric area** ($\times 10^{-2}$ μm^2)
PA1	66.5 ± 7.6	17.8 ± 3.1	96.6 ± 1.7	76.0 ± 7.1	52.8
PA2	60.6 ± 0.1	7.5 ± 0.8	92.3 ± 1.3	65.2 ± 0.8	52.8
PA3	61.4 ± 1.4	0.84 ± 0.01	68.6 ± 0.3	62.6 ± 0.9	52.8

Supplementary Table 6. Skeletonization statistics for the six PA membranes belonging to three synthesis conditions.

	Number of Branches	Number of Nodes	Terminal Nodes	Branching Nodes
PA1	2418	2338	1222	1116
	2275	2230	1224	1006
PA2	1807	1746	868	878
	1769	1680	808	872
PA3	1294	1230	592	638
	1485	1181	345	836

Supplementary Table 7. Additional GT parameters calculated for PA membrane skeletons.

Parameter	Formula	Definition
Mean degree of nodes	$k_i = e_i$<br>$k = \frac{\sum k_i}{v}$	The average number of branches connected to a given node. The simplest parameter describing a graph.[10]
Augmented vertex valence (*AVV*)<br>Augmented vertex sum (*AVS*)	$AVV_i = \sum_{i \neq j} \frac{k_j}{2^{l_{ij}}}$	*AVV* and *AVS* are used to quantify the complexity of a graph. *AVV* assigns a numerical value to each node of the graph by considering the degree of all nodes connected to the node of interest, weighted against the distance.[11]<br>*AVS* is the sum of *AVV* values of all the nodes of the graph with inequivalent symmetry.[12]
n : number of nodes<br>e : the number of branches<br>k : degree of a given node<br>l : distance between two nodes		

References

1 Kolev, V. & Freger, V. Hydration, porosity and water dynamics in the polyamide layer of reverse osmosis membranes: A molecular dynamics study. *Polymer* **55**, 1420–1426 (2014).

2 Li, N., Li, M., Lin, S., Cui, S. & Zhang, X. Stoichiometric effect on the structural transformation and spatial variation of polyamide reverse osmosis membranes: A molecular dynamics study. *J. Membr. Sci.* **686**, 121980 (2023).

3 He, J., McCutcheon, J. R. & Li, Y. Effect of different manufacturing methods on polyamide reverse-osmosis membranes for desalination: Insights from molecular dynamics simulations. *Desalination* **547**, 116204 (2023).

4 Shen, M., Keten, S. & Lueptow, R. M. Dynamics of water and solute transport in polymeric reverse osmosis membranes via molecular dynamics simulations. *J. Membr. Sci.* **506**, 95–108 (2016).

5 Muscatello, J., Müller, E. A., Mostofi, A. A. & Sutton, A. P. Multiscale molecular simulations of the formation and structure of polyamide membranes created by interfacial polymerization. *J. Membr. Sci.* **527**, 180–190 (2017).

6 Marbach, S. & Holmes-Cerfon, M. Mass Changes the Diffusion Coefficient of Particles with Ligand-Receptor Contacts in the Overdamped Limit. *Phys. Rev. Lett.* **129**, 048003 (2022).

7 Tan, Z., Chen, S., Peng, X., Zhang, L. & Gao, C. Polyamide membranes with nanoscale Turing structures for water purification. *Science* **360**, 518–521 (2018).

8 Karan, S., Jiang, Z. & Livingston, A. G. Sub–10 nm polyamide nanofilms with ultrafast solvent transport for molecular separation. *Science* **348**, 1347–1351 (2015).

9 An, H. *et al.* Mechanism and performance relevance of nanomorphogenesis in polyamide films revealed by quantitative 3D imaging and machine learning. *Sci. Adv.* **8**, eabk1888 (2022).

10 Vecchio, D. A., Mahler, S. H., Hammig, M. D. & Kotov, N. A. Structural Analysis of Nanoscale Network Materials Using Graph Theory. *ACS Nano* **15**, 12847–12859 (2021).

11 Randić, M. & Plavšić, D. On the concept of molecular complexity. *Croat. Chem. Acta* **75**, 107-116 (2002).

12 Nikolić, S., Trinajstić, N. & Tolić, I. M. Complexity of Molecules. *J. Chem. Inf. Comput. Sci.* **40**, 920–926 (2000)
