# Supplementary material for: Beyond nothingness in the formation and functional relevance of voids in polymer films
Source: Nat Commun. 2024 Apr 11;15:2852. doi: 10.1038/s41467-024-46584-2 (PMC11009415; doi:10.1038/s41467-024-46584-2)
Supplement: Supplementary file 3 — Description of Additional Supplementary Files [file 41467_2024_46584_MOESM3_ESM.pdf]

Description of Additional Supplementary Files

File Name: Supplementary Movie 1
Description: Grayscale intensity-based reconstructions of PA membranes.

File Name: Supplementary Movie 2
Description: Local thickness maps, void reconstructions and skeleton graphs for PA1, PA2 and PA3.

File Name: Supplementary Movie 3
Description: Cross-section (*xz*-slice) of a crumple region showing thin walls merging to form a thicker wall.

File Name: Supplementary Movie 4
Description: CGMD simulations showing the formation of a spanning membrane for PA3 and PA4.
